# Seven-year Safety and Efficacy of Somapacitan in Children With GH Deficiency: Final Results From REAL 3

**DOI:** 10.1210/jendso/bvaf189

**Published:** 2025-11-21

**Authors:** Lars Sävendahl, Tadej Battelino, Michael Højby, Tina Leunbach, Paul Saenger, Lawrence Silverman, Reiko Horikawa

**Affiliations:** Pediatric Endocrinology, Karolinska University Hospital and Department of Women's and Children's Health, Karolinska Institutet, Solna 171 77, Sweden; Faculty of Medicine, University of Ljubljana, and University Medical Center Ljubljana, Ljubljana 1000, Slovenia; Clinical Drug Development, Novo Nordisk A/S, Søborg 2860, Denmark; Clinical Drug Development, Novo Nordisk A/S, Søborg 2860, Denmark; Pediatric Endocrinology, NYU Langone Health, Mineola, NY 11501, USA; Pediatric Endocrinology, Goryeb Children's Hospital, Atlantic Health, Morristown, NJ 07962, USA; Division of Endocrinology and Metabolism, National Center for Child Health and Development, Tokyo 157-8535, Japan

**Keywords:** childhood growth hormone deficiency, growth hormone treatment, long-acting growth hormone, somapacitan

## Abstract

**Background:**

Somapacitan is a once-weekly GH treatment that has shown efficacy and safety profiles equivalent to daily GH in children with GH deficiency (GHD).

**Objective:**

To investigate long-term safety, efficacy, and treatment burden associated with somapacitan after 7 years (364 weeks) of treatment.

**Methods:**

REAL 3 (NCT02616562) was a phase 2, randomized, open-label trial investigating the efficacy and safety of somapacitan vs daily GH in children with GHD. After 156 weeks, participants entered a 208-week safety extension. Children in cohort I (age 2.5-10.0 years) previously completed 3 years in the trial; cohort II (age <2.5 years) and cohort III (age 9.0-17.0 years) entered the trial at week 156 for safety-only assessment. All participants received somapacitan 0.16 mg/kg/week. Height velocity (HV), HV SD score (SDS), height SDS, IGF-I SDS, incidence of adverse events (AEs), and treatment burden were assessed.

**Results:**

Overall, 43 participants (73%) in cohort I, 1 participant (100%) in cohort II, and 11 participants (69%) in cohort III completed the 208-week safety extension. Consistent increments in HV and HV SDS were seen in cohort I, and height SDSs at week 364 were close to 0. The incidence of AEs was aligned with previous investigations across all cohorts; 3 serious AEs in cohort I were considered probably/possibly related to treatment. Treatment burden was reduced in children who switched from daily GH to somapacitan.

**Conclusion:**

After 7 years of treatment, children with GHD receiving somapacitan experienced consistent increases in height SDS and reduced treatment burden. No new safety concerns were identified.

GH deficiency (GHD) is characterized by diminished growth, resulting in an adult height that is lower than expected according to the reference values [1]. Children with GHD are often treated with GH to enable them to attain adult height within the expected range [1]. Treatment can be administered as once-daily or once-weekly injections, and both options have demonstrated efficacy in increasing longitudinal growth in children with GHD and other non-GHD related disorders [2-5].

Somapacitan (Novo Nordisk A/S, Bagsvaerd, Denmark) is a once-weekly injectable GH treatment that is approved for children aged over 2.5 to 3 years and adults with GHD in the United States, European Union, and elsewhere [6, 7]. Reports on the efficacy and safety of somapacitan have been previously published [3, 8-11]. In the REAL 4 phase 3 trial, 200 treatment-naïve children with GHD were randomized to receive either somapacitan (0.16 mg/kg/week) or daily GH (0.034 mg/kg/day) for a 52-week main trial period, with results showing no statistically significant difference in height velocity (HV) between the 2 groups [8]. No new safety concerns were identified, and the observed safety profile of once-weekly somapacitan after 52 weeks of treatment was similar to the well-known profile of daily GH [8]. In addition, once-weekly somapacitan was shown to reduce treatment burden in comparison to daily GH [8], likely attributed to the lessened degree of distress and interference in daily life that is commonly associated with daily injections.

REAL 3 was a phase 2 trial designed to compare once-weekly somapacitan with daily GH (Norditropin®; Novo Nordisk A/S, Bagsvaerd, Denmark) in prepubertal children with GHD [8, 9]. REAL 3 results from the 26-week, 1-year, 2-year, 3-year, and 4-year data have been previously published [9-11]. Thus far, treatment with somapacitan has shown sustained efficacy and safety profiles and reduced disease burden [9-11], as well as clear preference for use by parents and guardians over daily GH [10]. Here, we report final safety, efficacy, and treatment burden results at year 7 (week 364) of the REAL 3 trial. These results represent the longest analysis of patients treated with somapacitan to date.

## Materials and Methods

### Study Design

REAL 3 (NCT02616562) was a phase 2, randomized, multinational, open-label, active-controlled, double blind, parallel-group, dose-finding trial designed to investigate the efficacy and safety of once-weekly somapacitan compared with daily GH (Norditropin FlexPro®) in GH-treatment-naïve, prepubertal children with GHD. Full details on the methodology of the trial have been previously published [10].

**Figure 1.**
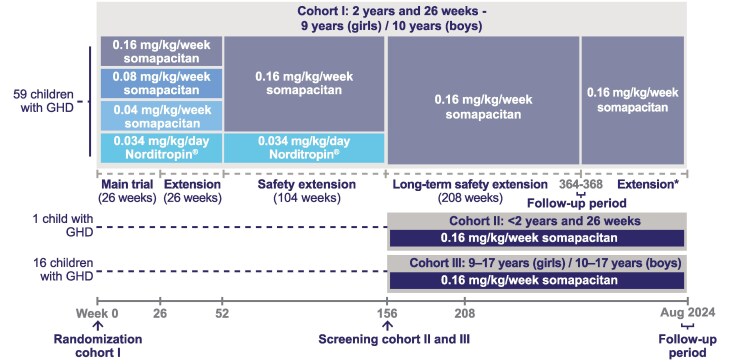
REAL 3 study design. REAL 3 (NCT02616562) was a phase 2, randomized, multinational, open-label, active-controlled, double-blinded, parallel group trial designed to investigate the efficacy and safety of once-weekly somapacitan compared with daily GH (Norditropin FlexPro) in GH treatment-naïve prepubertal children with GHD. The trial consisted of a 26-week main period, followed by a 26-week extension period. Then a 104-week safety extension took place, followed by a 208-week long-term safety extension period, with an additional 4-week follow-up period at the end of the trial to collect data on AEs. The main and extension trial periods were designed as a 4-arm parallel group trial with 3 blinded dose levels (0.04 mg/kg, 0.08 mg/kg, 0.16 mg/kg) of once-weekly somapacitan treatment and 1 active control arm of daily GH (0.034 mg/kg/day; Norditropin FlexPro). During the 104-week safety extension period, there were 2-arm parallel groups receiving 0.16 mg/kg somapacitan or daily GH. At the start of the 208-week long-term safety extension (week 156-364 of the trial), 2 cohorts (cohort II and III) were enrolled in addition to cohort I. Cohorts II and III represented age groups below and above the age range of cohort I, respectively, and were included upon request from the US Food and Drug Administration to access safety. During this period, all participants in each cohort were treated with 0.16 mg/kg/week somapacitan. Full details on the methodology of the trial have been previously published [10]. *Corresponds only to participants continuing treatment until somapacitan was available for prescription in their country or August 2024 at the latest. Abbreviations: AE, adverse event; GHD, GH deficiency.

In brief, the trial consisted of a 26-week main period, followed by a 26-week extension period. A subsequent 104-week safety extension was followed by a 208-week long-term safety extension period, with an additional 4-week follow-up period at the end of the trial to collect data on adverse events (AEs). The main and extension trial periods comprised a 4-arm parallel group trial treated with 3 blinded dose levels (0.04 mg/kg, 0.08 mg/kg, and 0.16 mg/kg) of once-weekly somapacitan and 1 active control arm of daily GH (0.034 mg/kg/day; Norditropin FlexPro) randomized at 1:1:1:1 (Fig. 1) [10]. After 52 weeks, participants entered the 104-week safety extension period as a 2-arm parallel group: all participants on somapacitan were switched to the 0.16 mg/kg/week dose, while participants on daily GH continued unchanged. After 156 weeks, all participants, including those previously on daily GH, received somapacitan 0.16 mg/kg/week during a further 208-week long-term safety extension. At the start of this safety extension (week 156–364 of the trial), 2 cohorts (cohorts II and III) were enrolled in addition to cohort I for safety assessment only. Thus, week 156 is considered the baseline period for cohorts II and III. From week 156 onwards, all participants in each cohort were treated with 0.16 mg/kg/week somapacitan until 364 weeks (the end of the long-term safety extension) or longer if somapacitan was not available for prescription in their country (until August 2024 at the latest, noted as week 442).

The primary objective of the REAL 3 trial was to evaluate the efficacy of multiple-dose regimens of once-weekly somapacitan after 26 weeks of treatment in GH treatment-naïve prepubertal children with GHD, compared with once-daily administration of GH. Further details of the primary and secondary objectives and endpoints have been previously reported [9]. The final results from the REAL 3 trial reported here are of cohorts I, II, and III and include data up to week 364 (7 years) of the trial. Efficacy, safety, and observer-related outcomes were assessed at this timepoint, with further details outlined in the following sections.

The protocol was approved in accordance with local regulations by appropriate health authorities and by an independent ethics committee/institutional review board, with country-specific rules for each site and country. The trial was conducted in accordance with the International Conference on Harmonization Guidelines for Good Clinical Practice and the Declaration of Helsinki. Informed consent was obtained in writing from the parents (and/or the child's legally acceptable representative), and child assent was obtained as age-appropriate before the first study procedure.

### Patients

Inclusion/exclusion criteria for cohort I were previously published [9-11]. Key eligibility criteria for children in cohort I were girls age 2.5 to 9.0 years and boys age 2.5 to 10.0 years at screening. All children in cohort I were GH-naïve and prepubertal at the time of enrollment, corresponding to Tanner stage 1 for pubic hair and testis volume (<4 mL) in boys and Tanner stage 1 for pubic hair and breast development (ie, no palpable glandular breast tissue) in girls. Key eligibility criteria for cohort II were children aged under 2.5 years with a minimum weight of 5 kg at screening. Key eligibility criteria for cohort III were girls age 9.0 to 17.0 years and boys age 10.0 to 17.0 years at screening. For cohorts II and III, children who had previously been treated with GH were eligible to enroll. Cohorts II and III represented age groups below and above the age range of cohort I, respectively. These cohorts were included upon request from the US Food and Drug Administration to assess safety in other age groups, in addition to cohort I, for whom treatment may be relevant.

All children had a confirmed diagnosis of GHD, determined either by 2 different GH stimulation tests with a defined peak GH level of ≤7.0 ng/mL (cohort I and GH-naïve children in cohort III) or as judged by investigators according to local practice (cohort II and previously treated children in cohort III). Children in cohort III had a bone age of less than 14 years for girls and less than 16 years for boys. Children with any clinical abnormality likely to affect growth or the ability to evaluate growth (eg, inability to stand) were excluded from all cohorts. This included any chromosomal aneuploidies, genetic mutations, congenital abnormalities including skeletal malformations, children born small for gestational age, or children receiving concomitant treatment with medications that could affect growth.

### Treatment

During the long-term safety extension (week 156-364), all participants received somapacitan 0.16 mg/kg/week (open label). Treatment was administered subcutaneously with a pen injector and could be injected any time during the designated dosing day (once per week).

The maximum treatment duration for a child was 364 weeks or longer if somapacitan was not available for prescription in their country [until August 2024 at the latest (week 442)]. Adherence to treatment was assessed using e-diaries, where each participant was asked to record the dose and time of administration. Treatment adherence was assessed by checking the participants' e-diary recordings against prescribed doses.

### Efficacy

Efficacy outcomes from 1 to 4 years of somapacitan use have been previously reported [9-11]. The efficacy outcomes assessed during the safety extension period included HV, HV SD score (SDS), height SDS, IGF-I SDS, IGF binding protein-3 (IGFBP-3) SDS, and bone age progression. IGF-I sampling was conducted every 26 weeks following week 156, within 7 days of somapacitan dosing. Near adult height measures were also assessed, including near adult height SDS and mid-parent height. Near adult height was defined as (1) HV of <2.0 cm/year calculated over a period of at least 9 months and (2) bone age of ≥16.0 years for males and ≥14.0 years for females or, if bone age was not available, a chronological age of ≥17.0 years for males and ≥15.0 years for females.

The current results describe the efficacy outcomes for cohort I from week 156 (year 3) up to week 364 (year 7) of the trial. For cohort II and III, the efficacy outcomes pertaining to IGF-I SDS and IGFBP3 SDS for the duration of their enrollment (weeks 156-364) in the trial are reported. Additionally, the efficacy outcomes for all participants who completed the follow-up period after week 364 are presented [data are reported from the extension that lasted until somapacitan became available for prescription in their country (until August 2024 at the latest, noted as week 442)]. Change in body mass index (BMI) SDS was assessed as an exploratory outcome related to growth and reported herein using descriptive statistics.

### Safety

The safety evaluation was based on all participants who were exposed to treatment in cohorts I, II, and III. AEs are presented from week 156 up to week 364. AEs experienced prior to week 156 in cohort I have previously been reported [10, 11]. AEs were assessed using descriptive statistics and summarized by treatment, Medical Dictionary for Regulatory Activities (MedDRA) system organ class and MedDRA preferred term. For participants who switched from daily GH to somapacitan at week 156, AEs were assigned to the treatment group at the onset of the AE, and if the onset was after the switch to somapacitan, the AE was assigned to both daily GH and somapacitan. In addition, the occurrence of drug-specific and in vitro neutralizing antibodies was investigated by the study sponsor using a validated antibody binding assay with a polyclonal antibody positive control (H. Solberg—Novo Nordisk Cat# S001221/1, RRID:AB_3717418) and reported using descriptive statistics, as previously described in detail [11, 12]. Glucose metabolism parameters [changes in fasting plasma glucose (FPG) and glycated hemoglobin (HbA_1c_)] were also assessed.

### Treatment Burden Outcomes

Treatment burden was assessed using disease-specific questionnaires from weeks 156 to 364 for cohort I only. These were the Treatment Burden Measure-Child-GHD-Observer (TB-CGHD-O) and the Treatment Burden Measure-Child-GHD-Parent/Guardian (TB-CGHD-P) [13]. All questionnaires were completed by the participants' parents or legal guardians. The scores of the questionnaires ranged from 0 to 100, and a lower score indicated a lower burden.

### Statistical Analyses

The full analysis set and the safety analysis set consisted of all randomly assigned children who received at least 1 dose of treatment. At week 364, efficacy [all reported as mean (SD): height SDS, HV SDS, IGF-I SDS, IGFBP-3 SDS, bone age progression, and BMI SDS], safety, and treatment burden outcomes [mean (SD)] were analyzed using descriptive statistics. Of note, for participants reaching near adult height, the following variables were analyzed using descriptive statistics: near adult height SDS, change from baseline (week 0) to the year near adult height was reached in height SDS, mid-parental target height SDS, and index of genetic height potential (derived from mid-parental target height SDS and near adult height SDS). The descriptive statistics for safety analyses included the number and percentage of participants who experienced AEs, the number of events, and the corresponding event rate.

## Results

**Figure 2.**
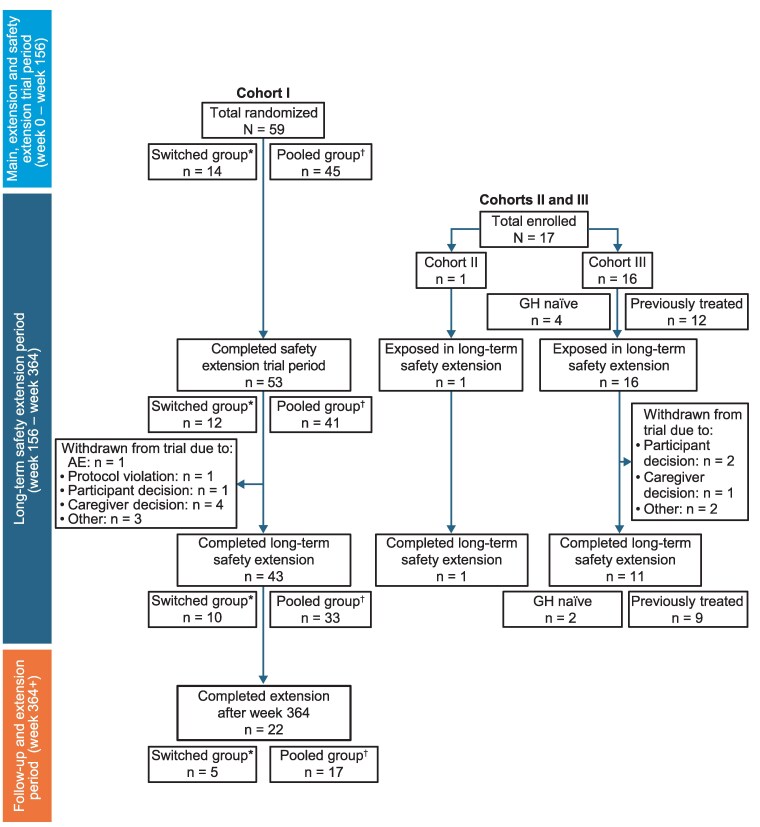
Participant flow diagram in the REAL 3 trial. *Norditropin 0.034 mg/kg/day/somapacitan 0.16 mg/kg/week. ^†^Somapacitan 0.16 mg/kg/week/somapacitan 0.16 mg/kg/week. Abbreviation: AE, adverse event.

A total of 43 out of the 59 participants in cohort I completed the long-term safety extension period (week 364; year 7), 10 of whom received daily GH from start of the trial up to week 156 and then switched to somapacitan 0.16 mg/kg/week (switched group) and 33 who received somapacitan throughout the trial (pooled group; Fig. 2). Overall, 22 participants from cohort I completed the follow-up period after the long-term safety extension and continued treatment after week 364, 5 of whom were in the switched group and 17 in the pooled group. One participant enrolled in cohort II, and 11 of the 16 participants enrolled in cohort III completed the long-term safety extension.

### Patient Disposition and Characteristics

Baseline demographics and characteristics are presented in Table 1. All children in cohorts I and II were prepubertal (Tanner stage 1) at screening, as per the inclusion criteria. In cohort III, 4 out of 4 GH-naïve children and 6 out of 12 previously treated children were prepubertal at baseline.

**Table 1.** Summary of baseline characteristics from the REAL 3 trial

	Cohort I		Cohort II	Cohort III	
	Norditropin/somapacitan (n = 14)	Somapacitan/somapacitan (n = 43)	Previously treated (n = 1)	Treatment naïve (n = 4)	Previously treated (n = 12)
Age (years)	5.9 (2.0)	5.9 (2.0)	2.50	12.9 (2.2)	12.7 (2.0)
Male (%)	64.3	58.1	100	75.0	91.7
Height (cm)	98.3 (13.8)	96.8 (13.7)	90.9 (−)	139.6 (9.6)	144.4 (16.7)
Body weight (kg)	15.5 (5.0)	14.4 (4.3)	13.2 (−)	44.0 (19.5)	38.6 (14.7)
HV, cm/year	3.7 (1.5)	4.3 (1.5)	—	6.2 (2.3)	7.7 (2.4)
HV SDS	−2.9 (2.1)	−2.4 (1.8)	—	0.2 (1.3)	1.3 (1.5)
HSDS	−3.4 (1.1)	−3.8 (1.8)	−0.1 (−)	−2.1 (0.6)	−1.4 (1.0)
IGF-I SDS	−2.1 (0.7)	−2.3 (0.9)	—	−0.7 (1.3)	0.1 (0.8)
BMI (kg/m^2^)	15.6 (1.4)	15.0 (1.1)	16.0 (−)	21.8 (6.9)	18.0 (2.4)
BMI SDS	−0.2 (0.9)	−0.6 (0.9)	−0.2 (−)	0.5 (1.5)	−0.3 (0.8)
GH peak (µg/L)	4.0 (2.0)	3.5 (2.2)	NA	4.1 (2.0)	4.7 (2.3)
Mother’s height (cm)	155.5 (9.4)	155.7 (6.8)	—	—	—
Father’s height (cm)	169.4 (8.7)	170.1 (8.3)	—	—	—

Data are mean (SD) unless otherwise specified.

Abbreviations: BMI, body mass index; HSDS, height SD score; HV, height velocity; n, number of participants; NA, not available; SDS, SD score.

For the switched group in cohort I, the total exposure to treatment was 38.2 years to daily GH and 43.9 years to somapacitan. For the pooled group, total exposure to somapacitan was 287.0 years. For the participant in cohort II, total exposure to somapacitan was 4.0 years, and for those in cohort III, total exposure was 37.7 years (10.2 and 27.5 years for GH-naïve and previously treated groups, respectively). Total patient-years of exposure to somapacitan 0.16 mg/kg/week specifically was 367.0.

In cohort I, mean adherence to treatment was 87.2% for daily GH (n = 14) between weeks 0 and 156; 83.7% at week 156 when they switched to somapacitan (n = 11); and 85.1% for the pooled somapacitan group (n = 43; Table 2).

**Table 2.** Summary of adherence to treatment, cohort I

	Norditropin 0.034 mg/kg/day (week 0-156)	Norditropin 0.034 mg/kg/day/somapacitan 0.16 mg/kg/week (week 156-442)*^a^*	Somapacitan 0.04/0.08/0.16 mg/kg/week pooled*^b^*
Number of participants	14	11	43
Number of reported dosings	12 736	2331	14 005
Number of dosings in adherence	12 734	2179	13 884
Adherence according to diary*^c^*			
Mean (SD)	87.2 (29.9)	83.7 (22.3)	85.1 (21.7)
Median	99.2	95.9	94.3
Min; max	4.1; 99.9	26.3; 98.6	0.0; 99.1

Dosings in adherence: all injections recorded between visit 2 and visit 38 in the diary with a dose above 0. A dose for Norditropin is counted in adherence if taken at or after 3 Am and before 3 Am the following day. A dose of somapacitan is counted in adherence if taken within 2 days before or 2 days after planned date of dosing.

^a^Including the long-term extension period, follow-up period, and subsequent extension period [lasting until somapacitan became available for prescription in their country (until August 2024 at the latest, noted as week 442)].

^b^The somapacitan (0.04/0.08/0.16 mg/kg/week) pooled arm contains all participants randomized to somapacitan in cohort I.

^c^Number of reported dosings from diary is observed dosing visits divided by number of planned dosings multiplied by 100.

### Efficacy

#### HV, HV SDS, and height SDS

**Figure 3.**
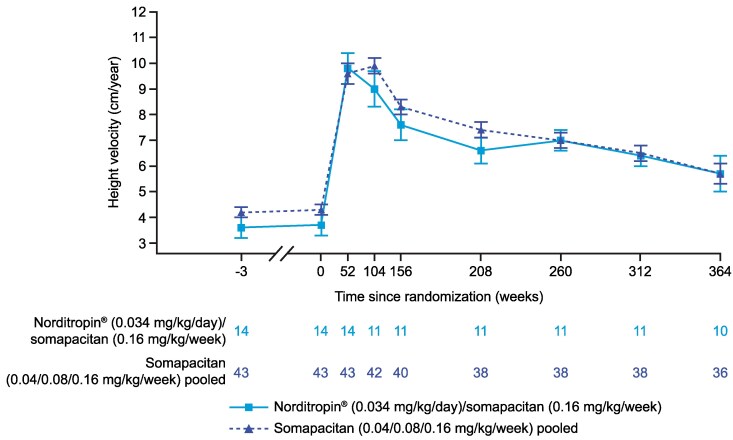
Mean HV (cm/year) from start of the trial up to week 364 for cohort I in the REAL 3 trial. Data are presented as mean (SE). At weeks 52 and 104, treatment groups are Norditropin 0.034 mg/kg/day only or pooled somapacitan. HV at week 156 is using height at week 104 as a reference. HV after week 156 to and including week 208 are using height at week 156 as a reference. HV after week 208 to and including week 260 are using week 208 as a reference. HV after week 260 to and including week 312 are using week 260 as a reference. HV after week 312 to and including week 364 are using week 312 as a reference. If height was not available at the reference visit, the last measurement prior to that was used. For participants whose final mean height at a visit showed a decrease compared with the previous visit, their tallest height has been carried forward for all height-based endpoint calculations. Abbreviation: HV, height velocity.

Similar trends in HV were observed from week 156 to week 364 for the 2 treatment groups in cohort I (Fig. 3). At week 156, mean (SD) HV was 7.6 (2.0) cm/year for the switched group and 8.3 (1.7) cm/year for the pooled group. At week 364, mean (SD) HV was 5.7 (2.2) cm/year for both switched and pooled groups.

**Figure 4.**
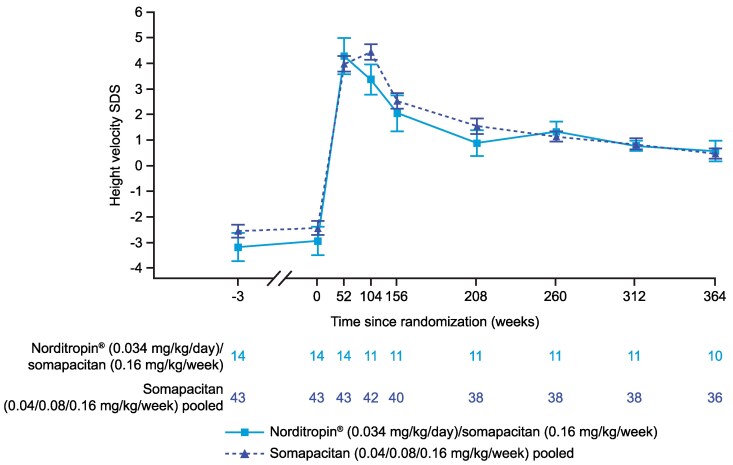
Mean HV SDS from start of the trial up to week 364 for cohort I in the REAL 3 trial. Data are presented as mean (SE). At weeks 52 and 104, treatment groups are Norditropin 0.034 mg/kg/day only or pooled somapacitan. For participants whose final mean height at a visit showed a decrease compared with the previous visit, their tallest height has been carried forward for all height-based endpoint calculations. Abbreviations: HV, height velocity; SDS, SD score.

Consistent changes in HV SDS were observed in line with HV (cm/year) within the switched and pooled groups in cohort I (Fig. 4). At week 156, mean (SD) HV SDS was 2.5 (1.6) for the pooled group and 2.1 (2.4) for the switched group. Mean (SD) HV SDS was 0.5 (1.1) and 0.6 (1.2) in the pooled group and switched group for cohort I at week 364, respectively.

**Figure 5.**
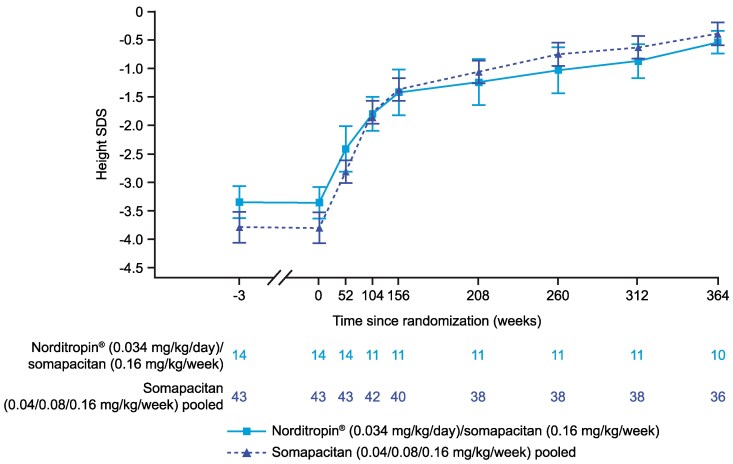
Mean HSDS from the start of the trial up to week 364 for cohort I in the REAL 3 trial. Data are presented as mean (SE). At weeks 52 and 104, treatment groups are Norditropin 0.034 mg/kg/day only or pooled somapacitan. For participants whose final mean height at a visit showed a decrease compared with the previous visit, their tallest height has been carried forward for all height-based endpoint calculations. Abbreviation: HSDS, height SD score.

Similar increments in height SDS were observed from week 156 to week 364 for the switched and pool groups in cohort I. At week 156, mean (SD) height SDS between the switched and pooled groups in cohort I was −1.42 (1.18) and −1.37 (1.24), respectively. Height at week 364 was within the ±2 SDS range for both treatment groups; mean (SD) height SDS was −0.5 (0.8) for the switched group and −0.4 (1.2) for the pooled group (Fig. 5).

#### Follow-up and extension period after week 364 (up to week 442)

Efficacy results during the follow-up and extension period (after week 364) were consistent with results up to week 364. At week 377, the mean (SD) HV for patients in the switched (n = 5) and pooled (n = 17) treatment groups of cohort I was 7.0 (2.5) and 5.7 (2.0) cm/year, respectively. At week 390, mean (SD) HV was 6.3 (1.7) cm/year in the switched group (n = 5) and 5.2 (1.5) cm/year in the pooled group (n = 11). At week 377, mean (SD) HV SDS was 1.2 (1.9) in the switched group and 0.4 (2.0) for the pooled group, and mean (SD) height SDS was −0.4 (1.0) and −0.2 (1.1) for the respective groups. At week 390, mean (SD) HV SDS was 0.7 (0.8) in the switched group and −0.02 (1.0) in the pooled group, and mean (SD) height SDS was −0.4 (1.0) and −0.1 (1.3), respectively.

#### Near adult height

In total, 2 participants in cohort I reached near adult height before week 364: 1 girl age 14.7 years and 1 boy age 16.0 years. Height measured at the visit at which these patients reached near adult height was 158.4 cm (−0.5 SDS) and 167.0 cm (−0.9 SDS), respectively. The mid-parental height (±8.0 cm) was 161.6 cm (−0.3 SDS) for the girl but was not available for the boy. One participant in cohort I reached near adult height after week 364. At week 390, 1 girl reached a height of 168.1 cm (+0.9 SDS) at 15.3 years of age. Her mid-parental height (±8.0 cm) was 160.0 cm (−0.4 SDS). Mean (SD) mid-parental height SDS for the girls with available data in cohort I was −0.3 (0.1).

#### Bone age progression

Bone age to chronological age ratio results showed similar advancement from weeks 156 to 364 in both treatment groups in cohort I. At week 156, the mean (SD) ratio was 0.9 (0.2) for the pooled group and 0.7 (0.2) for the switched group. At week 364, the mean ratio of bone age to chronological age was close to 1 for both groups [mean (SD): 1.0 (0.2)].

#### IGF-I SDS

**Figure 6.**
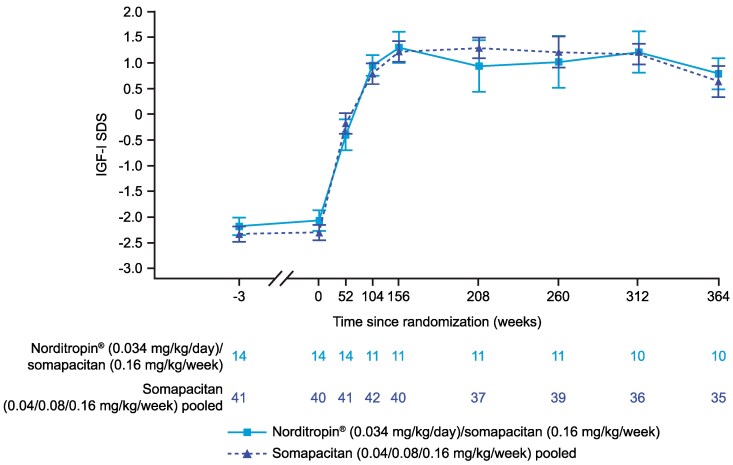
Mean IGF-I SDS from the start of the trial up to week 364 for cohort I in the REAL 3 trial. Data are presented as mean (SE). At weeks 52 and 104, treatment groups are Norditropin 0.034 mg/kg/day only or pooled somapacitan. Abbreviation: SDS, SD score.

IGF-I SDS was consistent in the 2 treatment groups in cohort I from week 156 to week 364. At week 364, mean (SD) IGF-I SDS values were 0.8 (1.0) for the switched group and 0.6 (1.7) for the pooled group (Fig. 6). For the participant in cohort II, IGF-I SDS remained consistent throughout the safety extension period; IGF-I SDS was 1.6 prior to enrollment (week 153) and 1.7 at week 364. For participants who were treatment-naïve in cohort III, mean (SD) IGF-I SDS at baseline (week 156) was −0.7 (1.3) and increased to 0.3 (0.9) by week 208 (week 364 of the trial). For the previously treated participants in cohort III, mean (SD) IGF-I SDS remained stable in the first year following treatment initiation, but variation was seen after 52 weeks of treatment (week 208 of the long-term safety extension period) in some participants in this group. Importantly, the mean IGF-I SDS were within the targeted reference range ±2 SDS for most participants across the 3 cohorts throughout the long-term safety extension period.

#### IGFBP-3 SDS

For cohort I, mean (SD) IGFBP-3 SDS remained in the range of ±2 from week 156 to week 364 (Table S1) [14]. At week 364, mean (SD) IGFBP-3 SDS was −0.3 (0.8) for the switched group and −0.2 (1.1) for the pooled group. The change in IGFBP-3 SDS from baseline to week 364 was comparable between the 2 treatment groups in cohort I, with a mean (SD) change of 1.7 (0.9) and 1.8 (1.1) for the switched and pooled groups, respectively. For the treatment-naïve participants in cohort III, mean (SD) IGFBP-3 SDS was −0.03 (1.8) at week 156 (n = 2), increased to 0.8 (0.5) at week 260 (n = 2), and decreased to −0.3 (0.5) by week 364 (n = 4). For previously treated participants in cohort III, mean (SD) IGFBP-3 SDS remained constant throughout the long-term safety extension period.

For participants in cohort I who completed the long-term safety extension period, IGFBP-3 SDS was consistent with the results at week 364. Mean (SD) IGFBP-3 SDS at week 390 was 0.1 (1.1) for the switched group and −0.5 (1.1) for the pooled group.

#### BMI SDS

Mean BMI SDS remained within the normal range (−2 to +2) across the 3 cohorts from week 156 to week 364. For cohort I, mean (SD) change in BMI SDS from baseline (week 0) to week 156 was −0.1 (2.0) in the switched group and 0.6 (0.9) in the pooled group. At week 364, mean (SD) change in BMI SDS from baseline was 0.5 (1.2) and 0.7 (1.0) in the switched and pooled groups, respectively.

For the participant in cohort II who entered the study at week 156, mean change in BMI SDS from week 156 to week 364 was 1.6. For treatment-naïve and previously treated participants in cohort III, who also entered the study at week 156, mean (SD) change in BMI SDS from week 156 to week 364 was −0.1 (1.1) and 0.4 (0.6), respectively.

### Safety

**Figure 7.**
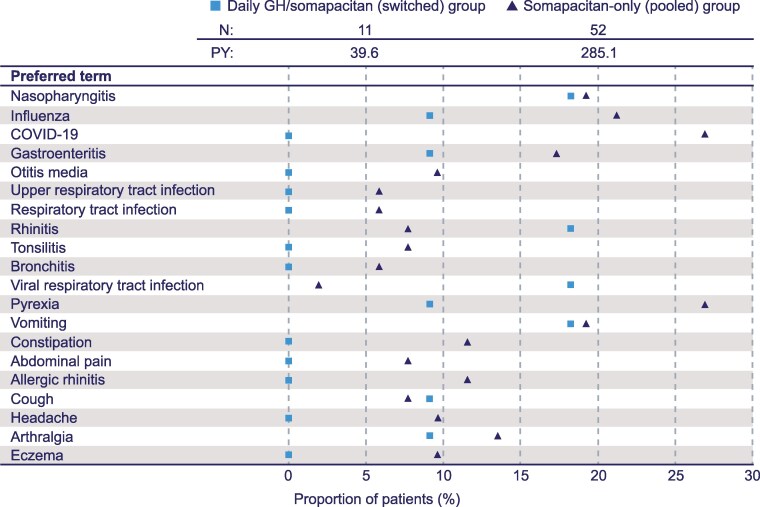
Most frequent AEs (occurring in ≥10% of patients) from week 0 to week 364 for cohort I in the REAL 3 trial. The somapacitan-only group contains all participants from cohort I from the day they received somapacitan 0.16 mg/kg/week. Abbreviations: AE, adverse event; N, number of patients; PY, total patient years at risk.

For cohort I, the rate of AEs for the switched group was 211.7 AEs/100 patient-years of exposure (PYE) prior to the switch and 247.7 AEs/PYE after switching to somapacitan at week 156. Rate of AEs for the pooled group was 191.2 AEs/10 PYE. The most frequent AEs (≥10%) for cohort I were common diseases occurring in children, such as nasopharyngitis, pyrexia, influenza, and gastroenteritis (Fig. 7). There was no clinically significant difference in the type and frequency of AEs reported between the switched and pooled groups (Table 3).

**Table 3.** Summary of AEs and SAEs in the REAL 3 trial

	Cohort I				Cohort II	Cohort III	
	Norditropin 0.034 mg/kg/day (week 0-156) (n = 14)	Norditropin 0.034 mg/kg/day (week 0-156)/somapacitan 0.16 mg/kg/week (week 156-364) (n = 11)	Somapacitan 0.04/0.08/0.16 mg/kg/week pooled*^a^* (week 0-364) (n = 45)	Somapacitan 0.16 mg/kg/week pooled*^a^* (week 0-364) (n= 52)	Previously treated (n = 1)	Treatment naïve (n = 4)	Previously treated (n = 12)
Total patient-years at risk	38.4	39.6	274.9	285.1	4.0	10.4	27.9
All AEs	14 (100.0)	10 (90.9)	43 (95.6)	50 (96.2)	1 (100.0)	2 (50.0)	9 (75.0)
SAEs	2 (14.3)	0	7 (15.6)	7 (13.5)	1 (100.0)	0 (0.0)	0 (0.0)
Severity							
Mild	14 (100.0)	9 (81.8)	42 (93.3)	46 (88.5)	1 (100.0)	2 (50.0)	9 (75.0)
Moderate	5 (35.7)	4 (36.4)	21 (46.7)	23 (44.2)	1 (100.0)	1 (25.0)	3 (25.0)
Severe	1 (7.1)	0	5 (11.1)	4 (7.7)	0	0	0
Causality (related to Norditropin)							
Probable	2 (14.3)	0	0	0	—	—	—
Possible	2 (14.3)	1 (9.1)	0	0	—	—	—
Unlikely	14 (100.0)	9 (81.8)	0	9 (17.3)	—	—	—
Causality (related to somapacitan)							
Probable	0	1 (9.1)	7 (15.6)	7 (13.5)	0	0	0
Possible	0	0	12 (26.7)	12 (23.1)	0	1 (25.0)	1 (8.3)
Unlikely	0	10 (90.9)	43 (95.6)	49 (94.2)	1 (100.0)	2 (50.0)	9 (75.0)
Action taken to trial product due to AE							
Drug interrupted	2 (14.3)	2 (18.2)	5 (11.1)	3 (5.8)	0	0	2 (16.7)
Drug withdrawn	2 (14.3)	0	1 (2.2)	1 (1.9)	0	0	0
Dose reduced	0	0	0	0	0	0	0
Dose increased	0	0	0	0	0	0	0
Dose not changed	14 (100.0)	10 (90.9)	43 (95.6)	49 (94.2)	1 (100.0)	2 (50.0)	9 (75.0)
Not applicable	0	0	4 (8.9)	4 (7.7)	0	1 (25.0)	3 (25.0)
Unknown	0	0	1 (2.2)	1 (1.9)	0	0	1 (8.3)
Outcome							
Recovered/resolved	14 (100.0)	9 (81.8)	43 (95.6)	48 (92.3)	1 (100.0)	2 (50.0)	9 (75.0)
Recovering/resolving	0	1 (9.1)	3 (6.7)	3 (5.8)	0	0	0
Recovered/resolved with sequelae	0	0	1 (2.2)	1 (1.9)	0	0	0
Not recovered/not resolved	7 (50.0)	6 (54.5)	23 (51.1)	27 (51.9)	1 (100.0)	1 (25.0)	4 (33.3)
Fatal	0	0	0	0	0	0	0
Unknown	0	0	0	0	0	0	0

Only AEs with an onset after the first administration of trial product and up until 14 days after last trial drug administration for withdrawn participants and with an onset after the first administration of trial product and up until visit 32 (cohort I: week 364; cohorts II and III: week 208) or 14 days after last trial drug administration, whichever comes first, for all other participants, are included. AE causality is based on the judgment of investigators.

Abbreviations: AE, adverse event; n, number of participants; SAE, serious adverse event.

^a^The somapacitan (0.04/0.08/0.16 mg/kg/week) pooled arm contains all participants randomized to somapacitan in cohort I. The somapacitan (0.16 mg/kg/week) pooled arm contains all participants from cohort I from the day they receive somapacitan 0.16 mg/kg/week.

The reporting rate of AEs that were probably/possibly related to treatment was 18.2 AEs/100 PYE for the switched group and 13.8 AEs/100 PYE for the pooled group. There was a total of 15 serious AEs (SAEs) reported in cohort I, most of which were moderate in severity (Table S2) [14]. At the time of the current analysis, 2 SAEs remained unresolved, and 1 SAE was under resolution. Three SAEs reported in the pooled group were considered probably/possibly related to the treatment and included epiphysiolysis, generalized edema, and vomiting. In cohort I, a total of 3 AEs [1 SAE of nephrotic syndrome and 2 AEs (drug hypersensitivity and arthralgia)] led to permanent discontinuation, and a total of 15 events led to temporary interruption of treatment. There were no AEs leading to dose reduction of the treatment in the entire duration of the trial. The single participant in cohort II experienced 2 SAEs assessed as unlikely to be related to treatment. These SAEs were norovirus infection and bronchitis due to respiratory syncytial virus; both were of moderate severity and were resolved.

In cohort III, the reporting rate of AEs in treatment-naïve participants was 115.4 AEs/100 PYE and 196.9 AEs/100 PYE in previously treated participants. No SAE was reported in cohort III.

Positive antibody tests were detected in 5 participants in cohort I and 1 participant who was treatment-naïve in cohort III. However, all tests were negative for in vitro neutralizing antibodies.

Glucose metabolism remained relatively stable in all 3 groups over the course of the long-term safety extension. In cohort I, at week 156, mean (SD) FPG was 5.0 (0.6) mmol/L in the switched group and 5.0 (0.5) mmol/L in the pooled group. At week 364, mean (SD) FPG levels remained the same for the switched group at 5.0 (0.6) mmol/L but decreased slightly for the pooled group to 4.8 (0.5) mmol/L. Mean (SD) HbA_1c_ at week 156 was 5.4 (0.2) % and 5.4 (0.3) % for the switched and pooled groups, respectively. At week 364, mean (SD) HbA_1c_ was 5.4 (0.3) % for both the switched group and the pooled group. For the participant in cohort II, mean FPG was 4.8 mmol/L prior to enrollment and decreased slightly to 4.2 mmol/L at week 364, while HbA_1c_ remained relatively stable (5.4% prior to enrollment and 5.6% at week 364).

Similar patterns for FPG and HbA_1c_ were seen in the treatment-naïve and previously treated groups of cohort III. Mean (SD) FPG prior to enrollment was 5.2 (0.3) mmol/L for the treatment-naïve group and 5.1 (0.3) mmol/L for the previously treated group. At week 364, mean (SD) FPG decreased to 4.6 (0.1) mmol/L and 4.7 (0.5) mmol/L in the treatment-naïve and previously treated groups, respectively. Mean (SD) HbA_1c_ values prior to enrollment were 5.4 (0.2) % and 5.3 (0.2) % for treatment-naïve and previously treated participants, respectively. At week 364, mean (SD) HbA_1c_ was 5.4 (0.1) % for treatment-naïve participants and 5.4 (0.3) % for those who were previously treated.

### Treatment Burden Outcomes

#### TB-CGHD-O and TB-CGHD-P

**Figure 8.**
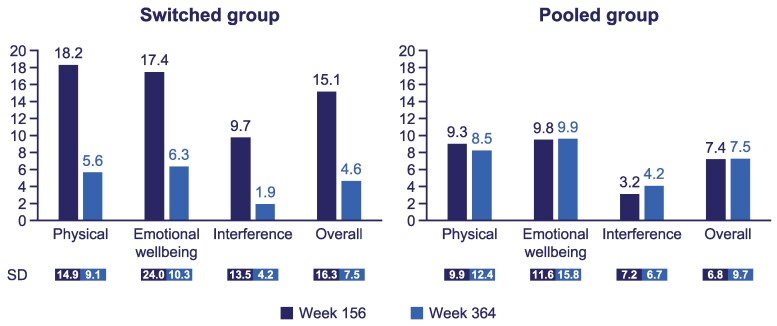
TB-CGHD-O scores from week 156 to week 364 for cohort I in the REAL 3 trial. Disease-specific questionnaires were conducted for cohort I only. All questionnaires were completed by the participants' parents or legal guardians. For the switched group, questionnaires were completed prior to the switch from daily GH to somapacitan at week 156. The scores range from 0 to 100, and a lower score indicates a lower burden. Abbreviation: TB-CGHD-O, Treatment Burden Measure-Child-GHD-Observer.

**Figure 9.**
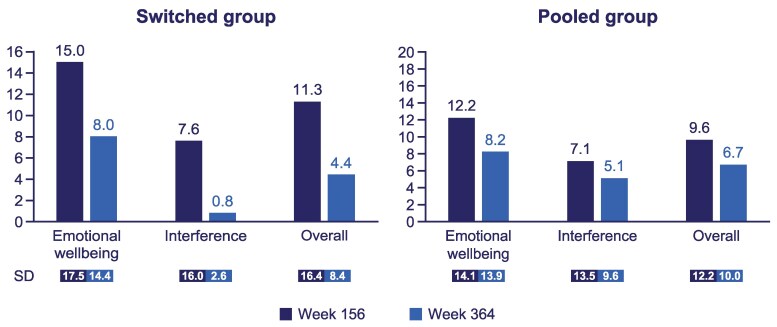
TB-CGHD-P scores from week 156 to week 364 for cohort I in the REAL 3 trial. Disease-specific questionnaires were conducted for cohort I only. All questionnaires were completed by the participants' parents or legal guardians. For the switched group, questionnaires were completed prior to the switch from daily GH to somapacitan at week 156. The scores range from 0 to 100, and a lower score indicates a lower burden. Abbreviation: TB-CGHD-P, Treatment Burden Measure-Child-GHD-Parent/Guardian.

Mean (SD) overall TB-CGHD-O score reduced from 15.1 (16.3) to 4.6 (7.5) for the switched group (those who switched from daily GH treatment to once-weekly somapacitan) and remained similar from 7.4 (6.8) to 7.5 (9.7) in the pooled group between weeks 156 and 364 (Fig. 8). For the parent/guardian perspective assessed with the TB-CGHD-P, the mean (SD) total score also reduced from 11.3 (16.4) to 4.4 (8.4) for the switched group and remained comparable from 9.6 (12.2) to 6.7 (10.0) for the pooled group between weeks 156 and 364 (Fig. 9).

## Discussion

The REAL 3 trial was designed to investigate the long-term efficacy and safety of somapacitan in children with GHD compared with daily GH. The long-term safety extension was implemented to obtain further insight into the efficacy and safety of long-term somapacitan treatment specifically in a broader range of age groups for whom treatment may be relevant. Final results at week 364 (year 7) support previously reported results of the trial [10, 11]; somapacitan effectively improved height outcomes and showed a similar tolerability and safety to known profiles of daily GH treatment. Importantly, patients in cohort I obtained a height SDS that was close to 0 for both treatment groups, indicating an increase in longitudinal growth and an improvement in height-based outcomes with somapacitan up to 7 years.

The safety profile of somapacitan in children with GHD is known from the previously published results of the REAL 3 trial [9-11]. At week 364, the rate of AEs/PYE for the switched group at week 156 of the trial (when they switched to somapacitan) was higher than that for the pooled group (entire trial duration) (247.7 vs 191.2 AEs/PYE). Overall treatment exposure to somapacitan for the switched group was 43.9 years and 287.1 years for the pooled group, indicating that the length of treatment with once-weekly GH does not impact the incidence of AEs. During the REAL 4 trial, the rate of AEs was 232.3 PYE in participants receiving somapacitan 0.16 mg/kg/week (n = 132) and 212.8 PYE in those receiving daily GH (n = 68) after 52 weeks of treatment [8]. Most AEs were mild and deemed unlikely related to study product. After all participants in the REAL 4 trial switched to somapacitan and completed treatment up to week 156, the rate of AEs reduced to 190.0 PYE in the pooled somapacitan group (n = 127) and 171.3 in the switched group (n = 67) [15]. Again, most AEs were mild in severity and considered unlikely related to the study product. In the results presented herein, no new safety or tolerability issues were identified after 364 weeks (7 years) of treatment with somapacitan. These results corroborate those seen previously [8, 10, 11], and it can be inferred that the safety profile of once-weekly somapacitan is similar to the profile observed for daily GH.

IGF-I levels are commonly used for monitoring the effect of GH treatment and in clinical practice as surrogate markers for compliance to GH treatment [16]. In our study, IGF-I levels increased following initiation of treatment in both treatment groups of cohort I but remained within the normal range (±2 SDS). In cohorts II and III, IGF-I levels remained relatively constant and within the normal range, but some participants in cohort III exhibited increases in IGF-I levels after the first year of treatment. There is an association between IGF-I levels and the pubertal growth spurt during which IGF-I levels peak [17]. The slight decrease in IGF-I SDS at week 364 seen in cohort I could be explained by the patients passing beyond the pubertal growth spurt. Indeed, after the maximal height velocity achieved during puberty, serum IGF-I levels remain elevated for approximately 2 to 3 years and slowly decline throughout adulthood [18]. This corresponds with the timing of peak HV at week 156 for participants in cohort I and the start of IGF-I level decline in the same group after week 312. The variation in IGF-I levels in cohort III after 52 weeks of treatment may also be attributed to the pubertal growth phase. Most participants in cohort III were male, and after 1 year of treatment, the mean age of participants would fall between 13 and 14 years, which aligns with the peak HV in males during the prepubertal and pubertal period [19]. In addition, if the participants had not yet started puberty before treatment initiation, they may have experienced a greater pubertal height gain because of catch-up growth, as reflected by the increase in IGF-I levels [20].

Across height-based outcomes, the normalization of HV and HV SDS can be observed in participants in cohort I who were on GH treatment for 7 years. HV SDS were consistently above 0, indicating an above-average growth rate. From week 156 to week 364, both switched and pooled treatment groups in cohort I showed similar growth patterns and demonstrated continuously increased height SDS for up to 7 years of treatment. These results suggest that changes in GH treatment regimens are unlikely to affect growth outcomes, even after several years of treatment. Indeed, in the REAL 4 trial, children with GHD who switched from daily GH to somapacitan had similar HV after 52 weeks of somapacitan treatment to those who continued somapacitan for 104 weeks (7.4 cm/year and 7.9 cm/year, respectively) [3]. After 156 weeks of treatment, mean (SD) annualized HV was 7.8 (1.4) cm/year in the switched group and 7.4 (1.5) cm/year in those continuing treatment with somapacitan, and mean (SD) change in height SDS from baseline to week 156 was 2.4 (1.1) and 2.0 (0.9), respectively [15]. However, previous evidence has suggested that baseline characteristics and baseline gene expression patterns are fundamental to treatment response, regardless of whether treatment is administered daily or once weekly [21]. In addition, pretreatment blood transcriptome analysis has been shown to be effective in predicting GH response, irrespective of GH treatment regimen [22]. During the long-term safety extension, it is likely that children in cohort I followed their genetic growth trajectory even when switching; thus it is possible that somapacitan and daily GH can exert similar effects on height attainment that are aligned with an individual's baseline characteristics and genetic treatment response.

Three children reached near adult height in this study and were age 14.7 (girl), 15.3 (girl), and 16.0 years (boy), 1 of whom received GH treatment for over 364 weeks (390 weeks). These children obtained their target height when available. This finding aligns with those from the REAL 4 trial, where participants reached a mean height SDS that was approaching mid-parental height SDS after 3 years of treatment, irrespective of whether they had switched from daily GH or received continued treatment with somapacitan [15]. An earlier start of GH treatment is associated with greater height outcomes and more likely achievement of near adult height within the genetic height potential [23]. These patients, all in cohort I, had a mean (SD) age of 5.9 (1.9) years at baseline. Thus, these patients had a longer period of prepubertal treatment ahead of them compared with some other participants, which may have increased the likelihood of near adult height attainment [23].

Treatment burden questionnaires were conducted from week 156 (year 3) of the trial in cohort I. At week 364 (year 7), the results showed that the group that received daily GH for 3 years and then switched to somapacitan experienced a considerable reduction in treatment burden, as observed in the participants as well as reported by the parents/guardians. For participants who received somapacitan for the duration of the trial, treatment burden seemed to be consistent. These results are consistent with the previously reported REAL 3 results and are likely attributable to the reduced injection frequency associated with once-weekly GH administration compared with daily administration (switched group), as well as the familiarity of treatment administration for longer periods of time (pooled group) [10]. In the REAL 4 trial, participants treated with somapacitan and their caregivers reported lower treatment burden than those treated with daily GH, and the difference was statistically significant for caregivers [8]. Furthermore, 10/11 (90.9%) of parents/caregivers whose children switched from daily GH to somapacitan preferred somapacitan over daily GH, for reasons including fewer injections, reduced worry about remembering to administer injections, and their child being less annoyed about having injections [3]. Similar results were found during REAL 6, a phase 3 GHD trial in China, wherein child and parent treatment burden assessments favored somapacitan over daily GH across physical, interference, emotional, and total score domains. The difference between treatments was statistically significant for parents/caregivers [21].

After 7 years, some participants (n = 16, 28.1%) in cohort I discontinued treatment or withdrew from the REAL 3 trial; the amount of missing data was low in all treatment groups, and overall adherence to treatment was high, lending credibility to the observed results. Additionally, participants with a broad range of demographics and baseline characteristics were included in this global trial. However, as adherence to treatment was assessed using e-diaries that were filled out by participants or their caregivers, inaccuracies in data collection were possible. Furthermore, the low number of participants in cohorts II and III (particularly cohort II, which only included 1 participant) hinder the application of results to wider age groups. IGF-I samples were taken every 13 weeks during the main treatment phase (up to week 156) and every 26 weeks thereafter, which may have affected the ability to derive a similarly clear weekly average level for dose titration after week 156. Nevertheless, the results of this long-term safety extension support previous results of the REAL 3 trial and provide evidence for the long-term efficacy and safety of once-weekly somapacitan for the treatment of children with GHD. The rationale for a reduced IGF-I sampling frequency pre- and postweek 156 was based on the previously published 3-year data from the study and careful monitoring of AEs that may have required dose adjustments based on IGF-I profile, none of which occurred.

To conclude, after 7 years of treatment, children with GHD who only received somapacitan experienced a consistent increase in height SDS. For children who switched from daily GH to somapacitan at week 156 (year 3) of the trial, height remained at the same trajectory. For most participants, IGF-I levels were within the normal range (±2 SDS) throughout the long-term safety extension period, and treatment burden questionnaires indicated that a once-weekly injection routine posed a reduced burden compared with a once-daily injection. The safety and tolerability profile observed for somapacitan after 7 years of treatment was comparable to the safety profile of daily GH and supports the previously reported safety results for shorter-term treatment duration.

## Data Availability

Some or all data sets generated and/or analyzed during the current study are not publicly available but are available from the corresponding author on reasonable request.

## Clinical Trial Information

Clinical trial registration: ClinicalTrials.gov NCT02616562 (registered November 25, 2015).
